# Vaginal *Lactobacillus* Detection and Urinary Findings During Synbiotic Supplementation in Pregnancy: An Exploratory Prospective Study

**DOI:** 10.3390/jpm16100513

**Published:** 2026-09-30

**Authors:** Maria Magdalena Montt-Guevara, Letizia Cardinali, Chiara Rossi, Lucrezia Finocchiaro, Francesca Tremolanti, Amerigo Ferrari, Elena Pisacreta, Tommaso Simoncini, Paolo Mannella

**Affiliations:** 1 Division of Obstetrics and Gynecology, Department of Clinical and Experimental Medicine, University of Pisa, 56126 Pisa, Italy; maria.montt@unipi.it (M.M.M.-G.); l.cardinali3@studenti.unipi.it (L.C.); l.finocchiaro2@studenti.unipi.it (L.F.); f.tremolanti@studenti.unipi.it (F.T.); amerigo.ferrari@santannapisa.it (A.F.); e.pisacreta@studenti.unipi.it (E.P.); tommaso.simoncini@unipi.it (T.S.); 2 Department of Surgical, Medical and Molecular Pathology and Critical Care Medicine, University of Pisa, 56126 Pisa, Italy; chiara.rossi@unipi.it

**Keywords:** vaginal microbiota, synbiotic, probiotics, *Lactobacillus*, pregnancy, urinalysis, personalized medicine

## Abstract

**Background/Objectives:** The vaginal and urinary tracts form an interconnected urogenital ecosystem. Oral probiotics have shown benefit in pregnancy for selected outcomes, including bacterial vaginosis and Group B *Streptococcus agalactiae* (GBS) colonization. However, their effects on vaginal *Lactobacillus* composition appear to be strain-specific. This prospective exploratory single-arm study evaluated whether vaginal *Lactobacillus* detection and routine urinary parameters changed during 60 days of oral multi-strain synbiotic supplementation in pregnant women with abnormal urinalysis. **Methods:** Twenty pregnant women at 28–32 weeks of gestation with abnormal routine urinalysis (leukocyturia, hematuria, and/or reported bacterial flora) received a multi-strain synbiotic containing seven probiotic strains and inulin twice daily for 60 days. Vaginal detection of *L. crispatus*, *L. reuteri*, *L. rhamnosus*, *L. gasseri* (supplement species), and *L. jensenii* (comparator) was assessed by species-specific real-time PCR at baseline (T0) and after 60 days (T1). Routine urinalysis was recorded at both times, and GBS colonization status at T1. Paired comparisons used McNemar’s exact and Wilcoxon tests; all analyses were exploratory. **Results:** Bacterial flora on urinalysis decreased from 20/20 (100%) to 8/20 (40%) (*p* < 0.001). Urinary pH, leukocyte and epithelial cell counts did not change significantly. Vaginal *L. reuteri* detection increased (2/20 to 10/20; *p* = 0.008). Four women (20%) were GBS-positive at T1. **Conclusions:** During 60 days of oral multi-strain synbiotic supplementation, temporal changes were observed in reported urinary bacterial flora and vaginal detection of selected *Lactobacillus* species. These exploratory findings require confirmation in larger controlled studies.

## 1. Introduction

The vaginal microbiota plays a central role in maintaining maternal urogenital health during pregnancy. In healthy pregnant women, it is typically dominated by *Lactobacillus* species [1]. These bacteria help maintain an acidic vaginal environment through the production of lactic acid and other antimicrobial compounds, thereby limiting the growth of potentially pathogenic microorganisms and protecting against ascending genital infections [2,3,4,5,6].

Despite the predominance of *Lactobacillus* species during normal pregnancy, vaginal dysbiosis may persist or develop in a subset of women. Vaginal dysbiosis has been associated with bacterial vaginosis, urinary and genital tract infections, preterm birth, and premature rupture of membranes [7,8,9]. Consequently, strategies aimed at maintaining or restoring vaginal eubiosis during pregnancy are of considerable clinical interest.

Evidence supporting probiotic approaches has largely come from non-pregnant women. In this population, randomized trials and meta-analyses of orally and vaginally administered lactobacilli have reported benefits in preventing recurrent urinary tract infections and in treating or preventing bacterial vaginosis, although findings vary across formulations and strains [10,11,12,13]. Proposed mechanisms include competitive exclusion of pathogens, production of antimicrobial substances, and modulation of mucosal immunity. In pregnancy, however, randomized trials of oral lactobacilli have reported inconsistent effects on vaginal microbiota composition and on bacterial vaginosis, and the vaginal detection of orally administered strains varies widely between studies [14,15,16,17,18,19]. This heterogeneity is thought to reflect the strain-specific nature of probiotic responses, together with the substantial interindividual variability of the maternal urogenital microbiota, suggesting that microbiome characterization may become relevant when optimizing probiotic interventions during pregnancy [1,15].

A further reason to explore vaginal *Lactobacillus* dynamics in late pregnancy is colonization by Group B *Streptococcus* (*Streptococcus agalactiae*, GBS), which affects approximately 18% of pregnant women worldwide and is the main indication for intrapartum antibiotic prophylaxis [20]. Vaginal colonization by GBS has been inversely associated with the presence of *L. crispatus* [21,22]. Randomized trials of oral probiotics aimed at reducing GBS vaginal–rectal colonization have yielded inconsistent results [23,24] and a systematic review reported only a modest effect with substantial heterogeneity between studies [18].

The female urinary tract is closely interconnected with the vaginal ecosystem, and increasing evidence supports the concept of a unified urogenital microbiota. Consequently, changes in the vaginal microbial community may also be reflected in urinary findings [25]. While the relationship between vaginal microbiota and urinary tract health has attracted growing interest, few studies have investigated whether oral probiotic supplementation during pregnancy is associated with simultaneous changes in vaginal *Lactobacillus* detection and routine urinary parameters [17,25,26].

The aim of this prospective exploratory single-arm study was to describe changes in the vaginal detection of selected *Lactobacillus* species and in routine urinary parameters during 60 days of oral multi-strain synbiotic supplementation in pregnant women with abnormal urinalysis findings. As a secondary exploratory objective, urinary and vaginal findings were described according to third-trimester GBS colonization status.

## 2. Materials and Methods

### 2.1. Study Design

This exploratory, single-arm study was conducted as a part of the MICROBIO project (CEAVNO approval nº. 28468_Mannella), a prospective observational study at the Obstetrics and Gynecology Unit of Azienda Ospedaliero-Universitaria Pisana (Pisa, Italy). The study was designed to characterize changes in the vaginal microbiota during pregnancy. Women were eligible for enrollment from the first trimester onward and were recruited during routine antenatal visits at the outpatient clinic.

Within the MICROBIO cohort, women who presented with abnormal routine urinalysis findings at 28–32 weeks of gestation between June and December 2025 were offered oral synbiotic supplementation. These women constituted the population evaluated in the present sub-study. Paired vaginal swabs and routine urinalyses were obtained immediately before supplementation (T0) and after completion of the 60-day supplementation period (T1). Although the broader MICROBIO study included samples collected at earlier gestational ages, the present analysis was restricted to paired assessments obtained before and after synbiotic supplementation during the third trimester.

The sub-study had no untreated or placebo control group, and participants were not randomized. The MICROBIO study has not yet been published. All data presented here, including paired species-specific vaginal real-time PCR findings, routine urinalysis results, and GBS colonization status among supplemented women, are novel and have not been reported elsewhere.

The study was conducted in accordance with the Declaration of Helsinki and approved by the Comitato Etico Regione Toscana–Area Vasta Nord Ovest (CEAVNO; protocol code MICROBIO; approval No. 28468_Mannella, 22 May 2025). Written informed consent was obtained from all participants before enrollment.

### 2.2. Study Population and Eligibility Criteria

Eligible participants were women aged 18 years or older with a singleton spontaneous pregnancy of physiological course, between 28+0 and 32+0 weeks of gestation, who presented an abnormal routine antenatal urinalysis and provided written informed consent. An abnormal urinalysis was defined as the presence of leukocyturia (≥20 cells/µL), hematuria, and/or bacterial flora reported on microscopic examination of the urinary sediment.

Women with conditions known to affect the vaginal or intestinal microbiome, including gestational diabetes mellitus, pre-pregnancy BMI ≥ 30 kg/m^2^, autoimmune diseases, or inflammatory bowel disease, recurrent urinary tract infections (≥3 episodes in the previous year), or pregnancy complications (preterm premature rupture of membranes, preterm labor, fetal growth restriction, pre-eclampsia) were not eligible. Women who had received antibiotics or probiotics in the last 2 months preceding enrollment, or who had urinary symptoms requiring antibiotic treatment, were not considered for supplementation. Eligibility criteria are summarized in Table 1.

No formal sample size calculation was performed. The study included all eligible women identified during the recruitment period who accepted supplementation; the sample size therefore reflects the number of women available rather than a pre-specified target.

### 2.3. Intervention and Adherence

All participants received an oral multi-strain synbiotic supplement (Devamix^®^, Enteralia Bioscience, Milan, Italy) for 60 days, administered as one 2.5-g stick pack twice daily according to the study protocol. Each unit-dose pack contained a microencapsulated, gastro-resistant powder of live probiotic strains in an orodispersible formulation, which could be taken directly in the mouth or dissolved in water, preferably on an empty stomach or approximately 15 min before meals, and did not require refrigeration. The formulation contained seven probiotic strains, inulin as a prebiotic component, and bovine colostrum, as summarized in Table 2.

This formulation was selected because it combines several *Lactobacillus* species commonly represented in the healthy vaginal microbiota (including *L. crispatus*, *L. gasseri*, and *L. rhamnosus*) with a delivery technology designed to protect bacterial viability during gastric transit—a prerequisite for the proposed gut–vagina transfer route. The 60-day supplementation period was chosen so that the follow-up assessment (T1) would coincide with the routine third-trimester antenatal evaluation, which includes vaginal–rectal GBS screening.

Adherence to supplementation was assessed by participant self-report at the follow-up visit (T1); no stick counts or supplement diaries were used. Tolerability and the occurrence of adverse events were also assessed by self-report at the same visit.

### 2.4. Study Timeline and Data Collection

The present analysis was based on the two visits relevant to the supplementation period. Women were identified as eligible on the basis of an abnormal routine antenatal urinalysis. After providing informed consent, baseline characteristics were collected: including maternal age, pre-pregnancy BMI, parity, smoking status, and dietary habits (assessed with a brief study-specific questionnaire covering major food groups and water intake). At the baseline visit (T0), performed between 28+0 and 32+0 weeks of gestation immediately before starting supplementation, a vaginal swab was collected for DNA extraction and qPCR analysis, and the results of the routine antenatal urinalysis were recorded. Participants were instructed to repeat the routine urinalysis upon completion of the supplementation period. The follow-up visit (T1) took place after completion of the 60-day supplementation period, at approximately 36–41 weeks of gestation, depending on the gestational age at the time of enrollment. At T1, a second vaginal swab was collected, the results of the repeat urinalysis were recorded, and vaginal–rectal screening for Group B Streptococcus (GBS) was performed during the same visit as part of standard third-trimester obstetric care.

### 2.5. Vaginal DNA Extraction and Real-Time PCR Analysis

#### 2.5.1. Selection of Target Species

Five vaginal *Lactobacillus* species were selected a priori for species-specific analysis. Four species included in the synbiotic formulation—*L. crispatus*, *L. rhamnosus*, *L. gasseri*, and *L. reuteri*—were evaluated to explore whether their species-level vaginal detection changed during oral supplementation. *L. jensenii*, a common member of the healthy vaginal microbiota that was not included in the formulation, was assessed as a non-supplement reference species to contextualize the observed temporal changes. These analyses were exploratory and were not intended to demonstrate gut-to-vagina transfer, vaginal colonization by the administered strains, or participant adherence. Because the assays were species-specific rather than strain-specific, they could not distinguish the DSM strains contained in the supplement from endogenous strains of the same species.

#### 2.5.2. DNA Extraction and Real-Time PCR

Vaginal swab specimens were collected using eNAT^®^ Collection and Preservation System (Copan Italia S.p.A., Brescia, Italy). Total bacterial DNA was extracted using the PureLink™ Microbiome DNA Purification Kit (Cat. No. A29790; Thermo Fisher Scientific, Waltham, MA, USA) according to the manufacturer’s instructions. DNA concentration and purity were assessed using a NanoDrop spectrophotometer (Thermo Fisher Scientific), after which the extracted DNA was stored at −20 °C until analysis.

The presence of vaginal *Lactobacillus crispatus* (Ba04646245_s1), *L. reuteri* (Ba07922708_s1), *L. rhamnosus* (Ba07922683_s1), *L. gasseri* (Ba04646234_s1), and *L. jensenii* (Ba04646258_s1) was assessed by TaqMan real-time PCR using species-specific assays (Thermo Fisher Scientific) on a QuantStudio™ 3 Real-Time PCR System (Thermo Fisher Scientific). The bacterial 16S rRNA gene (Ba04230899_s1) was used as an endogenous control in all samples. Each reaction was performed in duplicate in a final volume of 20 µL containing 9 ng of template DNA and TaqMan™ Fast Advanced Master Mix (Cat. No. 4444557; Thermo Fisher Scientific). Amplification was performed using the manufacturer’s recommended fast cycling conditions (50 amplification cycles), and amplification data were analyzed using QuantStudio™ Design & Analysis Software (RUO), version 3.0 (Thermo Fisher Scientific).

Ct values were not available for all observations because several samples showed no amplification for one or more target species. Results were therefore analyzed qualitatively, as the presence or absence of each target species, rather than by Ct-based quantification. Samples showing a characteristic amplification curve were classified as positive (species detected. Detection rates were expressed as the number and percentage of positive participants at each study visit.

A sample was classified as negative (species not detected) only when two conditions were met. First, both duplicate reactions showed no amplification for the target species; for every sample and target species, the two replicates yielded identical results, and no sample showed amplification in only one replicate. Second, the 16S rRNA endogenous control amplified successfully in the same sample, confirming the presence of amplifiable bacterial DNA and excluding technical amplification failure. A negative result was therefore interpreted as the absence of the target species rather than as a failed reaction.

To minimize inter-assay variability, DNA extraction, quantification, and real-time PCR analysis were performed only after both baseline (T0) and follow-up (T1) vaginal swabs had been collected, allowing paired samples from each participant to be processed within the same analytical batch.

The species-specific assays identify each target at the species level and do not discriminate between individual strains. Therefore, the detection of a given species cannot be attributed specifically to the DSM strains contained in the supplement, as it may also reflect endogenous strains of the same species.

### 2.6. Urinalysis and Group B Streptococcus Screening

Urine specimens were collected according to standard antenatal practice, with instructions to cleanse the genital area, discard the first portion of the urine stream, and collect a midstream sample (clean-catch technique). Specimen collection followed routine clinical procedures and was not additionally standardized for the purposes of this study. Urinalyses were performed at two local clinical laboratories, which applied the same reference thresholds for leukocyte counts and sediment parameters.

Urinalysis included chemical analysis and microscopic examination of urinary sediment. Urinary pH, leukocyte count, epithelial cell count, and the presence of bacterial flora were recorded. Leukocyturia was defined as ≥20 cells/µL and an elevated epithelial cell count as ≥28 cells/µL, according to the laboratory reference values. Bacterial flora was classified as absent, scarce, moderate, or abundant and was considered present whenever detected.

Urine cultures were not part of the study protocol and were not systematically performed; when requested by the treating physician as part of routine care, results w ere either negative or showed bacterial counts below 5 × 10^4^ CFU/mL. In the absence of clinically significant urinary symptoms, antibiotic treatment was not considered indicated. At the follow-up visit, participants were specifically asked about urinary symptoms; none reported symptoms requiring antibiotic treatment, and no participant received antibiotics during the supplementation period.

At T1, all participants underwent routine vaginal–rectal screening for Group B *Streptococcus* (GBS; *Streptococcus agalactiae*) as part of standard third-trimester obstetric care.

### 2.7. Statistical Analysis

Statistical analyses were performed using GraphPad Prism version 11.0 (GraphPad Software, Boston, MA, USA); McNemar’s exact tests were performed using the MedCalc online statistical calculator (MedCalc Software Ltd., Ostend, Belgium; version 23.7.1, https://www.medcalc.org/en/calc/mcnemar.php, accessed on 9 July 2026).

Continuous variables are presented as median and interquartile range (IQR), whereas categorical variables are presented as absolute frequencies and percentages. Paired comparisons between baseline (T0) and follow-up (T1) were performed using the Wilcoxon matched-pairs signed-rank test for continuous variables and McNemar’s exact test for paired categorical variables, including the detection of each *Lactobacillus* species and the presence of urinary bacterial flora. Bacterial flora was analyzed as a dichotomous variable (present/absent) because it is reported on routine urinalysis as a semi-quantitative category rather than as a numerical count. Leukocyte and epithelial cell counts were analyzed both as continuous variables and, additionally, as dichotomous variables according to the laboratory reference thresholds, whereas urinary pH was analyzed as a continuous variable only.

Given the small size of the GBS-positive subgroup (*n* = 4), comparisons of urinary and vaginal findings according to GBS colonization status were considered exploratory and are presented descriptively only; no inferential statistical analyses were performed.

All statistical tests were two-sided, and a *p*-value < 0.05 was considered statistically significant. Given the exploratory nature of the study, no formal adjustment for multiple comparisons was applied to the primary analyses, and all *p*-values should be interpreted as hypothesis-generating. As a sensitivity analysis, a Bonferroni correction accounting for the five *Lactobacillus* species tested was applied (adjusted α = 0.01).

No formal a priori sample size calculation was performed. The sample size was based on the number of eligible participants recruited during the study period and was considered appropriate for an exploratory analysis intended to generate preliminary data and inform the design of future adequately powered studies. Accordingly, the study was not powered to detect small between-visit differences, and non-significant findings should be interpreted with caution.

## 3. Results

### 3.1. Study Population

**Figure 1:**
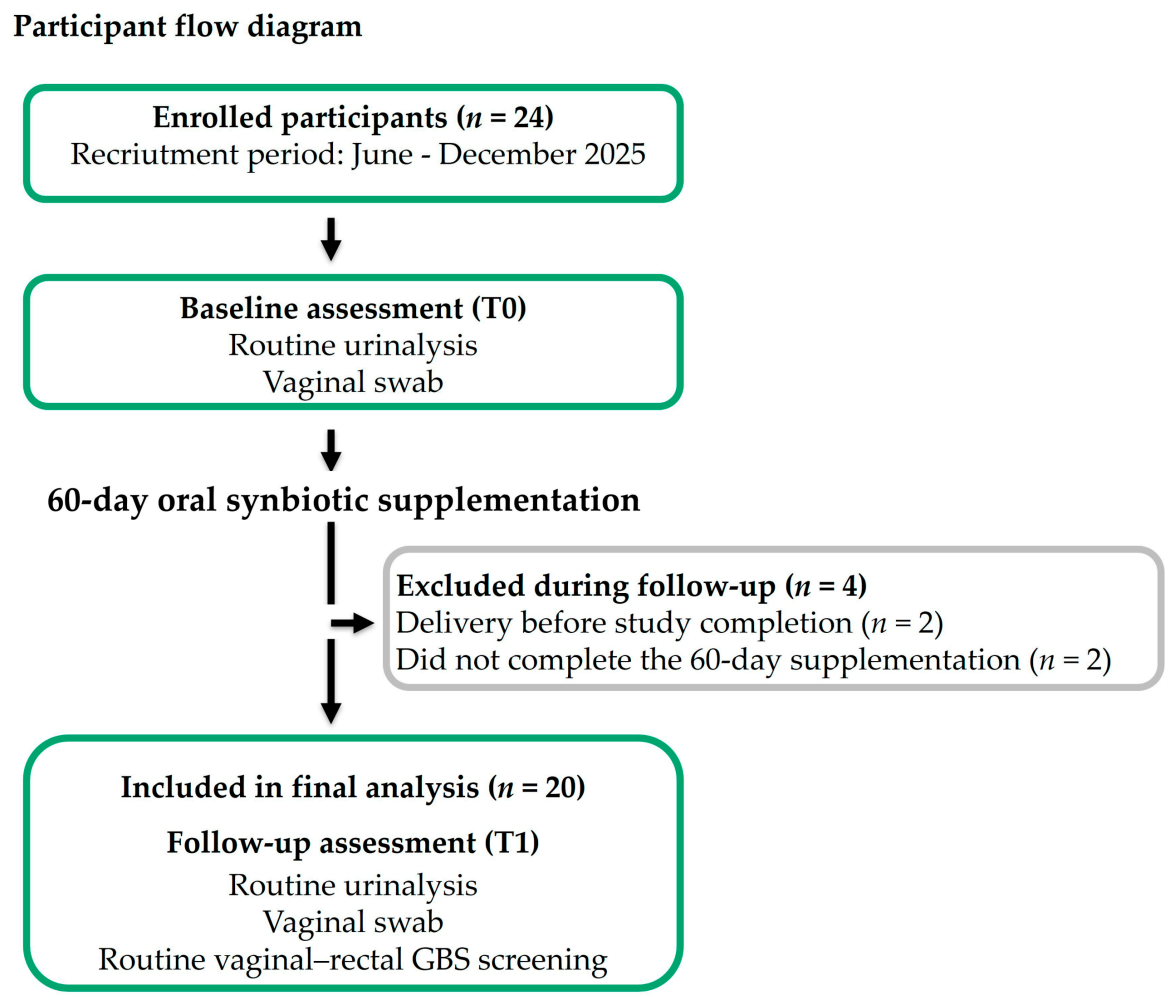
Participant flow diagram. Twenty-four pregnant women fulfilling the inclusion criteria were enrolled. Abnormal urinalysis was defined as leukocyturia (≥20 cells/µL), hematuria, and/or bacterial flora. After 60 days of oral synbiotic supplementation, four participants were excluded during follow-up, leaving 20 women for the final analysis. Baseline (T0) and follow-up (T1) assessments included routine urinalysis performed as part of standard antenatal care and vaginal swab collection, followed by DNA extraction and qPCR analysis of *Lactobacillus* species. Routine vaginal–rectal GBS screening was performed at T1 according to standard obstetric care.

A total of 24 pregnant women with an abnormal urinalysis at screening were enrolled between June and December 2025. Four participants were excluded during follow-up: two because delivery occurred before study completion, and two who discontinued supplementation after approximately 40 days for personal reasons, with no discontinuation attributed to adverse effects. The remaining 20 women completed the study protocol, including both baseline (T0) and follow-up (T1) assessments, and were included in the final analysis (Figure 1). Baseline characteristics of the 20 participants are summarized in Table 3. By design, no participant had received antibiotics or probiotics within the 2 months preceding enrollment.

No adverse events were reported during the supplementation period, and all 20 participants who completed the study reported adherence to the supplementation regimen.

### 3.2. Changes in Vaginal Species Detection During Supplementation

The 16S rRNA gene was successfully amplified in all 20 samples at both T0 and T1, confirming the adequacy of DNA extraction and the suitability of all samples for downstream analysis.

**Figure 2:**
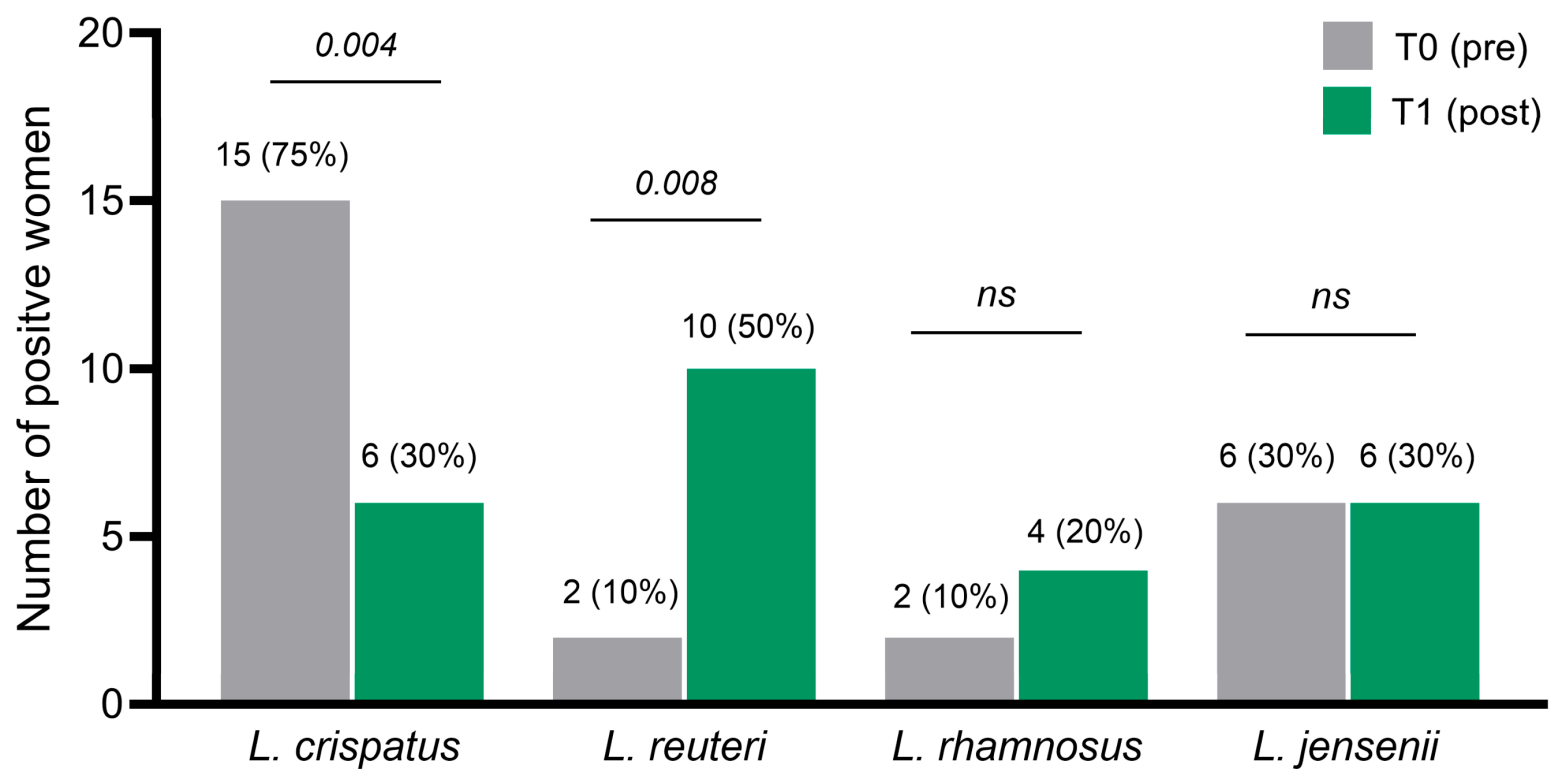
Detection rate of vaginal *Lactobacillus* species assessed by qPCR before (T0) and after (T1) 60 days of synbiotic supplementation (*n* = 20). Bars show the number and percentage of women positive for each species. *L. gasseri* was not detected in any sample at either time point (0/20 at T0 and T1) and is therefore not included in the graph. McNemar’s exact test; ns = not significant.

The detection of each target species in vaginal swabs was compared between T0 and T1 (Figure 2). Among the species contained in the supplement, two showed a significant change during the 60-day supplementation period. *Lactobacillus crispatus* decreased from 15/20 (75%) at T0 to 6/20 (30%) at T1 (9 losses, 0 gains; *p* = 0.004), whereas *L. reuteri* increased from 2/20 (10%) to 10/20 (50%) (8 gains, 0 losses; *p* = 0.008). In the Bonferroni-adjusted sensitivity analysis (α = 0.01), both changes remained statistically significant. The remaining supplement-contained species did not change significantly: *L. rhamnosus* was detected in 2/20 (10%) women at T0 and 4/20 (20%) at T1 (*p* = 0.625), while *L. gasseri* was not detected in any sample at either time point. *L. jensenii*, which is not a component of the administered supplement, remained stable throughout the study period (6/20, 30%, at both time points; *p* = 1.0).

### 3.3. Changes in Urinalysis Parameters During Supplementation

**Figure 3:**
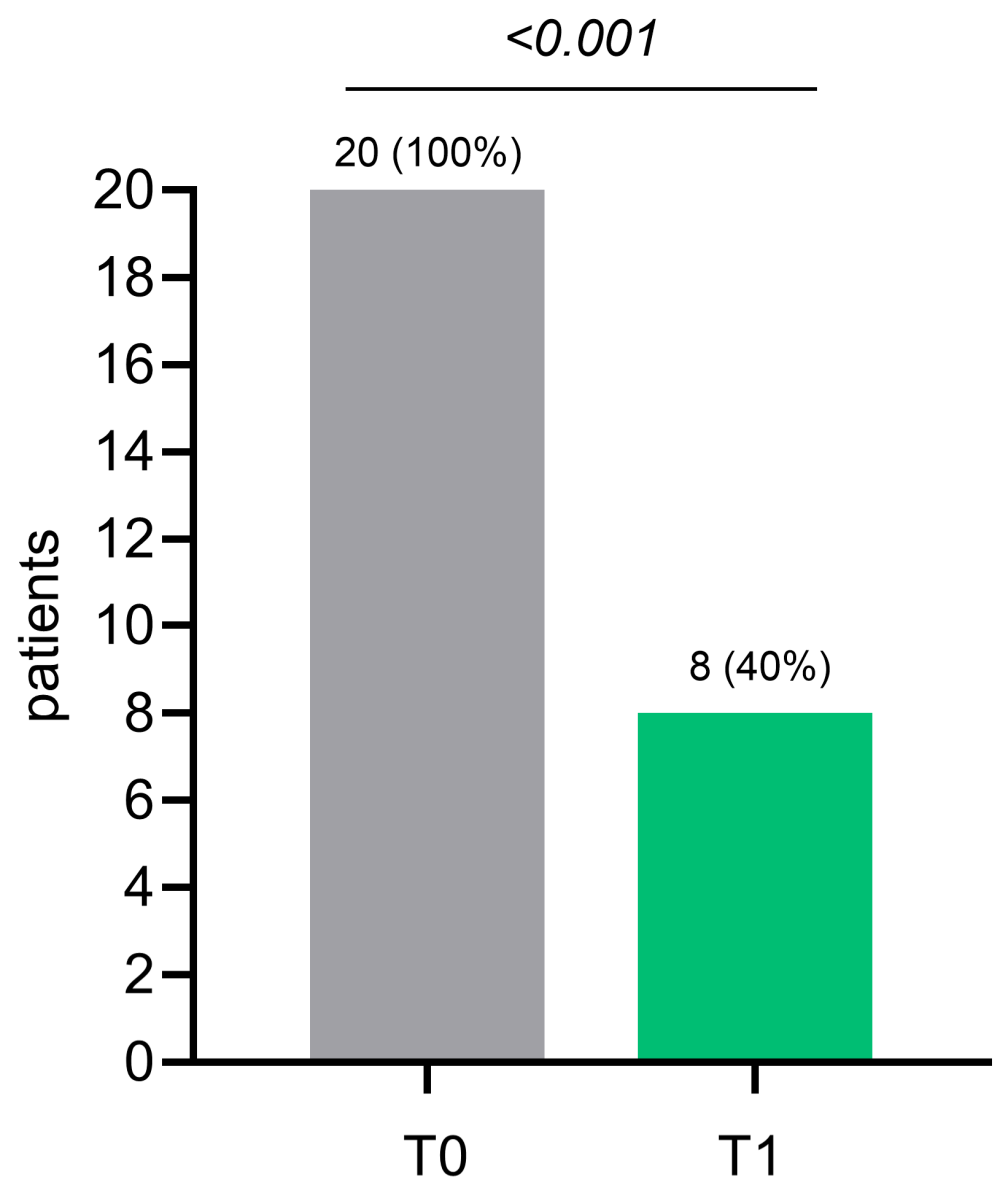
Prevalence of bacterial flora on urinalysis at baseline (T0) and after 60 days of synbiotic supplementation (T1). The prevalence decreased from 20/20 (100%) to 8/20 (40%) participants (McNemar’s exact test, *p* < 0.001).

At enrollment, all 20 participants presented bacterial flora on routine urinalysis. Following 60 days of oral synbiotic supplementation, the prevalence of bacterial flora was significantly reduced from 20/20 women (100%) at baseline (T0) to 8/20 women (40%) at follow-up (T1), representing an absolute reduction of 60 percentage points (*p* < 0.001) (Figure 3; Table 4).

In contrast, the other urinalysis parameters did not change significantly. Leukocyte counts were unchanged (median 10 vs. 7 cells/µL; *p* = 0.602), as was the proportion of women with elevated leukocytes (8/20 to 6/20; *p* = 0.727). Epithelial cell counts showed a non-significant downward trend (median 32 vs. 15.5 cells/µL; *p* = 0.178; elevated in 13/20 vs. 7/20; *p* = 0.113). Urinary pH was unchanged (median 6.5 at both time points; *p* = 0.641) (Table 4).

### 3.4. Group B Streptococcus Screening

At T1, all women underwent vaginal–rectal swabbing for Group B *Streptococcus* (GBS; *Streptococcus agalactiae*) as part of the routine third-trimester screening performed at the end of pregnancy. Screening was negative in 16/20 women (80%) and positive in 4/20 (20%).

### 3.5. Descriptive Analysis of GBS-Positive Women

Among the 4 women (20%) with a positive vaginal–rectal swab for GBS at T1, leukocyte counts worsened in 2, epithelial cell counts worsened in 2, and bacterial flora persisted in 2. None of the four GBS-positive women had detectable vaginal *L. crispatus* at T1, compared with 6 of 16 GBS-negative women.

By comparison, among GBS-negative women, worsening of leukocyte counts was observed in only 1 of 16 participants, no worsening of epithelial cell counts was observed, and bacterial flora cleared in 10 of 16 women.

Given the small size of the GBS-positive subgroup (*n* = 4), these observations are descriptive and hypothesis-generating only and were not subjected to inferential statistical testing.

## 4. Discussion

In this prospective exploratory single-arm study, we evaluated changes in vaginal *Lactobacillus* species detection and routine urinary parameters during 60 days of oral multi-strain synbiotic supplementation in pregnant women with abnormal urinalysis findings. Three principal observations emerged. First, the proportion of women with bacterial flora on routine urinalysis decreased substantially during supplementation, in the absence of antibiotic therapy. Second, changes in vaginal *Lactobacillus* detection were species-specific: *L. reuteri* detection increased significantly, whereas *L. crispatus* detection decreased. Third, the four women colonized with Group B *Streptococcus* (GBS) at the end of pregnancy showed persistence or worsening of urinary findings more frequently than GBS-negative participants, and none had detectable vaginal *L. crispatus* at follow-up, although these subgroup observations are descriptive only. Given the prospective exploratory single-arm design, these observations are hypothesis-generating; their biological plausibility and potential clinical implications are discussed below.

From a clinical perspective, the principal observation was the reduction in urinary bacterial flora, from 100% at baseline to 40% after 60 days of supplementation, without antibiotic treatment. Our cohort occupies a specific clinical niche. In routine antenatal care, three scenarios are possible for an asymptomatic woman undergoing urinalysis. In most pregnancies, urinalysis is normal, and only standard follow-up is needed. At the opposite extreme, culture-confirmed asymptomatic bacteriuria meets quantitative criteria, and antibiotic treatment is recommended by guidelines [27]. Between these extremes lies an intermediate scenario: asymptomatic women with an altered urinary sediment suggesting urogenital dysbiosis, in whom urine culture is not routinely performed and antibiotic treatment is not indicated. This is the population of our study. In this context, interest is growing in strategies that support the urogenital microbiota as a way to avoid unnecessary antibiotic exposure during pregnancy, and probiotic or synbiotic supplementation is being explored as one such approach [27,28]. Our study contributes preliminary data in a population—pregnant women with asymptomatic urinary abnormalities not requiring antibiotic treatment—in which this strategy had not been specifically evaluated.

However, this finding must be interpreted with caution, for two reasons. First, urine cultures were not systematically performed. Bacterial flora on routine urinalysis cannot diagnose bacteriuria or urinary tract infection, and we cannot know how many participants would have had culture-confirmed bacteriuria; in the subset with cultures requested as part of routine care, none met quantitative criteria for asymptomatic bacteriuria. For this reason, our findings should not change current clinical management. Second, an alternative explanation must be acknowledged: part of the reported bacterial flora may reflect the passage of urine through the urogenital environment rather than true bladder bacteriuria. Epithelial cells, commonly regarded as a marker of specimen contamination, were elevated in 13/20 women at baseline and in 7/20 at follow-up; differences in specimen collection between visits therefore cannot be excluded as a contributing factor. At the same time, because the urinary tract and vagina form a single interconnected urogenital environment, voided-urine findings in pregnancy inevitably reflect this continuum to some degree, and distinguishing “contamination” from genuine features of the urogenital ecosystem is inherently difficult with routine urinalysis. Despite these caveats, the hypothesis that supporting the urogenital microbiota could help avoid unnecessary antibiotics deserves testing in controlled studies with urine-culture endpoints.

This hypothesis is biologically plausible in light of increasing evidence that the vagina and urinary tract constitute a single interconnected urogenital ecosystem rather than two independent anatomical compartments. Thomas-White et al. demonstrated that the bladder and vagina share highly similar bacterial strains, including health-associated *Lactobacillus* species, supporting the concept of an integrated female urogenital microbiota [29]. Similarly, Gottschick et al. reported parallel alterations of the vaginal and urinary microbial communities in women with bacterial vaginosis [30]. Although routine urinalysis cannot characterize the urinary microbiome itself, the parallel changes observed in urinary bacterial flora and vaginal *Lactobacillus* detection are consistent with the concept that modulation of one compartment may influence the other.

Evidence regarding oral probiotic supplementation during pregnancy remains inconsistent. Randomized placebo-controlled trials using oral *L. reuteri* RC-14 and *L. rhamnosus* GR-1 generally failed to demonstrate significant modifications of the vaginal microbiota [14,16,17], and Yefet et al. did not detect *L. reuteri* RC-14 in vaginal samples following oral administration [15,19]. Conversely, administration of *L. rhamnosus* CA15 (DSM 33960) has been associated with an improvement in vaginal microbiota composition in women with dysbiosis [31]. These apparently conflicting observations support the concept that probiotic effects are largely strain-specific and cannot be generalized across different bacterial formulations. Differences in dose are unlikely to explain these discrepancies: the daily dose of the four *Lactobacillus* species assessed in our study (approximately 1.8 × 10^10^ cells) falls within the range used in previous pregnancy trials, from 1 × 10^9^ cells per strain in the EFFPRO trial [14] to 1 × 10^10^ in Yefet et al. [15,19]. Notably, the trial with the highest dose per strain reported the least favorable colonization outcome, suggesting that the specific strain, rather than the administered quantity, may be the main determinant of vaginal detection after oral intake. Direct comparison remains limited, however, by differences in strains, populations, and formulation, including microencapsulation [32].

In the present study, vaginal detection increased for both *L. reuteri* and *L. rhamnosus*, reaching statistical significance only for *L. reuteri*. The synbiotic formulation differed substantially from those evaluated in previous pregnancy studies, containing *L. reuteri* DSM 23878 and *L. rhamnosus* DSM 21981 instead of the widely investigated RC-14/GR-1 combination. Furthermore, microencapsulation may have enhanced bacterial survival during gastrointestinal transit [32], potentially increasing delivery of viable microorganisms to the gut. Two internal observations support the specificity of the observed changes. *L. jensenii*, not contained in the formulation, remained stable (30% at both time points), indicating that the changes did not involve the entire lactobacillar community. Conversely, *L. gasseri*, although included in the formulation, was not detected in any vaginal sample, confirming that oral administration does not translate uniformly into vaginal detection for every species.

The most unexpected finding was the reduction in vaginal detection of *L. crispatus*, despite this species representing the predominant bacterial component of the administered synbiotic formulation. At first glance, this observation may appear paradoxical. However, rather than considering it a negative finding, we believe it underscores the complexity of the vaginal microbial ecosystem and the ecological interactions occurring after administration of a multi-strain synbiotic.

Importantly, the objective of the present study was not to investigate the vaginal persistence or transfer of individual probiotic strains after oral administration. Instead, the clinical objective was to evaluate whether oral multi-strain synbiotic supplementation could be associated with changes in the vaginal ecosystem and urinary findings in pregnant women presenting abnormal urinalysis. All participants entered the study because of abnormal urinary findings, suggesting an altered urogenital microbial environment despite frequent baseline detection of *L. crispatus*. Consequently, a shift to microbial homeostasis should not necessarily be expected to produce a proportional increase in every administered bacterial species, but rather a broader ecological reorganization of the microbial community.

Several biological mechanisms may account for the observed reduction in *L. crispatus* detection. First, the vaginal microbiota is a dynamic ecosystem during pregnancy, in which fluctuations in the relative prevalence of individual *Lactobacillus* species may occur despite preservation of an overall lactobacilli-dominated community [2,6]. Second, different *Lactobacillus* species display distinct ecological behaviors, colonization dynamics and competitive interactions, making their response to oral probiotic supplementation inherently species-specific. Third, the formulation contained multiple bacterial species, which may have modified the ecological balance of the vaginal community without promoting a uniform increase in every constituent species. Finally, our molecular approach evaluated the presence or absence of selected bacterial species rather than their quantitative abundance. Therefore, the reduction in *L. crispatus* detection should not be interpreted as evidence of a true quantitative decline but may instead reflect changes in detectability or in the ecological distribution of *Lactobacillus* species within the vaginal microbial community. Whether these observations represent ecological redistribution of resident bacteria, pregnancy-related microbial dynamics, or differential adaptation of the administered species cannot be established from the present study.

Finally, we explored urinary and vaginal findings according to GBS colonization status at routine third-trimester screening. Vaginal–rectal GBS prevalence in the present study (20%) was consistent with the mean prevalence reported for European pregnant populations (approximately 19%) [20] and with rates observed in third-trimester screening cohorts [22]. Women colonized with GBS more frequently showed persistence or worsening of urinary findings, and none had detectable vaginal *L. crispatus* at follow-up. Although this sub-group comprised only four women, these observations are consistent with previous reports linking GBS colonization to reduced *L. crispatus* abundance during pregnancy [21,22]. Future prospective studies are needed to determine whether GBS colonization identifies women with a less resilient urogenital microbial ecosystem and whether synbiotic supplementation may influence this relationship.

Beyond the biological findings, our results may also have implications for future research on personalized approaches. The heterogeneous response observed among individual *Lactobacillus* species suggests that the response to supplementation might depend on the baseline composition of the urogenital microbial ecosystem—a hypothesis that could not be tested in the present study.

This study has several limitations. First, it was a single-center, prospective exploratory single-arm study with a limited sample size and was therefore not powered to detect small between-visit differences; without a control group, the observed changes cannot be distinguished from spontaneous temporal dynamics of the vaginal microbiota in late pregnancy. Second, participants were selected on the basis of abnormal urinalysis and acceptance of supplementation, which may introduce selection bias, although the strict eligibility criteria yielded a homogeneous cohort. Third, adherence was assessed by self-report only and could not be objectively verified. Dietary habits were assessed at enrollment using a simple non-validated questionnaire, and all participants reported a Mediterranean-style dietary pattern; however, dietary intake was not quantified or monitored during supplementation, so residual dietary variability cannot be excluded as a confounder. Fourth, the qPCR analysis was qualitative and species-specific. It assessed the presence or absence of each species but did not measure bacterial abundance, characterize the overall microbial community, or distinguish between strains. The increase in *L. reuteri* detection may reflect either the administered DSM 23878 strain or endogenous *L. reuteri*. Quantitative within-participant comparisons were not feasible because only six women were positive for *L. crispatus* at both time points and only two were positive for *L. reuteri*. Fifth, urinalysis was performed at two local laboratories using the same reference thresholds, but specimen collection followed routine clinical procedures and urine cultures were not systematically performed; as discussed above, reduced specimen contamination cannot be excluded as a contributing explanation for the decrease in reported bacterial flora. Finally, the GBS-positive subgroup was too small for inferential analyses. Nevertheless, the prospective paired design and the simultaneous assessment of both compartments of the urogenital axis in the same cohort provide preliminary, hypothesis-generating observations that warrant confirmation in larger randomized placebo-controlled trials.

## 5. Conclusions

In this prospective exploratory single-arm study, 60 days of oral multi-strain synbiotic supplementation in pregnant women with abnormal urinalysis was accompanied by a substantial reduction in reported urinary bacterial flora and by species-specific changes in the vaginal *Lactobacillus* detection: an increase in *L. reuteri* and a decrease in *L. crispatus*. These changes occurred without antibiotic treatment, in a clinically relevant but understudied population for whom no established management exists. These results are hypothesis-generating: the small sample size, the absence of a control group, and the qualitative nature of species detection do not allow causal inference. Our findings support the concept of an interconnected urogenital ecosystem and identify the urogenital axis as a possible target for future non-antibiotic strategies in pregnancy. Confirmation in randomized placebo-controlled trials with urine-culture endpoints and quantitative microbiome profiling is now needed.

## Figures and Tables

**Table 1:** Eligibility criteria for the study population.

Inclusion Criteria	Exclusion Criteria
Age ≥18 years Singleton spontaneous pregnancy of physiological course Gestational age between 28+0 and 32+0 weeks Abnormal routine antenatal urinalysis * Written informed consent Acceptance of oral synbiotic supplementation	Gestational diabetes mellitus Pre-pregnancy BMI ≥ 30 kg/m^2^ Autoimmune disease or inflammatory bowel disease Recurrent UTI (≥3 in the previous year) Pregnancy complications (PPROM, preterm labor, fetal growth restriction, or pre-eclampsia) Antibiotic or probiotic use in the previous 2 months Urinary symptoms requiring antibiotic treatment

* Defined as leukocyturia (≥20 cells/µL), hematuria, and/or bacterial flora reported on microscopic examination of the urinary sediment. BMI, body mass index; PPROM, preterm premature rupture of membranes; UTI, urinary tract infection.

**Table 2:** Composition of the multi-strain synbiotic supplement (Devamix^®^, Enteralia Bioscience, Milan, Italy). Doses are expressed as viable cells per 2.5-g stick and per day (two sticks daily). All bacterial strains were microencapsulated with a glyceric fatty-acid ester coating. DSM, Deutsche Sammlung von Mikroorganismen; LMG, Belgian Coordinated Collections of Microorganisms.

Species	Deposit No.	Unit-Dose Pack	Dose per Day
*Lactobacillus crispatus*	DSM 24619	5 × 10^9^ cells	10 × 10^9^ cells
*Lactobacillus acidophilus*	DSM 21717	2 × 10^9^ cells	4 × 10^9^ cells
*Lactobacillus rhamnosus*	DSM 21981	2 × 10^9^ cells	4 × 10^9^ cells
*Lactobacillus gasseri*	DSM 32405	1 × 10^9^ cells	2 × 10^9^ cells
*Lactobacillus reuteri*	DSM 23878	1 × 10^9^ cells	2 × 10^9^ cells
*Lactobacillus plantarum*	LMG P-21021	1 × 10^9^ cells	2 × 10^9^ cells
*Bifidobacterium longum*	DSM 16603	1 × 10^9^ cells	2 × 10^9^ cells
Inulin (prebiotic)	-	1000 mg	2000 mg
Bovine colostrum (55% globulins)	-	20 mg	40 mg

**Table 3:** Baseline characteristics of the study population (*n* = 20).

Characteristic	Value (*n* = 20)
Maternal age, years—median (IQR)	32 (26.3–35.0)
Pre-pregnancy BMI, kg/m^2^—median (IQR)	23.1 (21.0–24.8)
Nulliparous, n (%)	12 (60.0%)
Smoking during pregnancy, n (%)	0 (0%)
Mediterranean-style diet, n (%)	20 (100%)
Gestational age at T0, weeks—median (IQR)	29+1 (28+4–30+6)

IQR: interquartile range; BMI: body mass index; T0: baseline.

**Table 4:** Urinalysis parameters at baseline (T0) and after 60 days of synbiotic supplementation (T1) (n = 20). Categorical variables were compared using McNemar’s exact test and continuous variables using the Wilcoxon matched-pairs signed-rank test. Elevated leukocyte count was defined as ≥20 cells/µL and elevated epithelial cell count as ≥28 cells/µL according to laboratory reference values. Bacterial flora was classified as absent, scarce, moderate, or abundant and was considered present whenever any degree of flora was reported.

Parameter	T0	T1	*p*-Value
Bacterial flora present, n (%)	20 (100%)	8 (40.0%)	<0.001
Leukocytes, cells/µL—median (IQR)	10.0 (3.5–42.2)	7.0 (4.0–37.5)	0.602
Elevated leukocyte count (≥20 cells/µL), n (%)	8 (40.0%)	6 (30.0%)	0.727
Epithelial cells, cells/µL—median (IQR)	32.0 (7.0–71.7)	15.5 (3.7–58.2)	0.178
Elevated epithelial cell count (≥28 cells/µL), n (%)	13 (65.0%)	7 (35.0%)	0.113
Urinary pH—median (IQR)	6.5 (6.0–7.0)	6.5 (6.0–6.5)	0.641

## Data Availability

The data presented in this study are available on request from the corresponding author. The data are not publicly available due to privacy and ethical restrictions concerning sensitive clinical information from pregnant participants.
